# Fabrication of a Biomass-Derived Activated Carbon-Based Anode for High-Performance Li-Ion Batteries

**DOI:** 10.3390/mi14010192

**Published:** 2023-01-12

**Authors:** Faheem Ahmed, Ghazzai Almutairi, Prince M. Z. Hasan, Sarish Rehman, Shalendra Kumar, Nagih M. Shaalan, Abdullah Aljaafari, Adil Alshoaibi, Bandar AlOtaibi, Kaffayatullah Khan

**Affiliations:** 1 Department of Physics, College of Science, King Faisal University, P.O. Box 400, Al-Ahsa 31982, Saudi Arabia; 2 National Center for Energy Storage Technologies, King Abdulaziz City for Science and Technology (KACST), Riyadh 11442, Saudi Arabia; 3 Center of Nanotechnology, King Abdulaziz University, Jeddah 22254, Saudi Arabia; 4 Chemistry Department, McGill University, 801 Sherbrooke St. W, Montreal, QC H3A 0B8, Canada; 5 Department of Physics, School of Engineering, University of Petroleum & Energy Studies, Dehradun 248007, India; 6 Physics Department, Faculty of Science, Assiut University, Assiut 71516, Egypt; 7 Department of Civil and Environmental Engineering, College of Engineering, King Faisal University, Al-Ahsa 31982, Saudi Arabia

**Keywords:** biomass, activated carbon, Li-ion batteries, XRD, TEM

## Abstract

Porous carbons are highly attractive and demanding materials which could be prepared using biomass waste; thus, they are promising for enhanced electrochemical capacitive performance in capacitors and cycling efficiency in Li-ion batteries. Herein, biomass (rice husk)-derived activated carbon was synthesized via a facile chemical route and used as anode materials for Li-ion batteries. Various characterization techniques were used to study the structural and morphological properties of the prepared activated carbon. The prepared activated carbon possessed a carbon structure with a certain degree of amorphousness. The morphology of the activated carbon was of spherical shape with a particle size of ~40–90 nm. Raman studies revealed the characteristic peaks of carbon present in the prepared activated carbon. The electrochemical studies evaluated for the fabricated coin cell with the activated carbon anode showed that the cell delivered a discharge capacity of ~321 mAhg^−1^ at a current density of 100 mAg^−1^ for the first cycle, and maintained a capacity of ~253 mAhg^−1^ for 400 cycles. The capacity retention was found to be higher (~81%) with 92.3% coulombic efficiency even after 400 cycles, which showed excellent cyclic reversibility and stability compared to commercial activated carbon. These results allow the waste biomass-derived anode to overcome the problem of cyclic stability and capacity performance. This study provides an insight for the fabrication of anodes from the rice husk which can be redirected into creating valuable renewable energy storage devices in the future, and the product could be a socially and ethically acceptable product.

## 1. Introduction

Nowadays, the market demand for energy storage devices is growing continuously due to fast growth of electric vehicles, wearable electronics, and smartphones, which attracted researchers to focus on the development of novel energy storage devices. Since the first commercialization of Li-ion batteries in 1991 by Sony, due to their excellent properties including high energy density, outstanding cyclic stability, and astonishing storage capacity, Li-ion batteries are in high demand [1,2,3]. In order to evaluate the advantages of batteries, various performance indicators, which include the production cost, energy storage capacity density, rate performance, lifetime, and cyclic stability, are used [4,5,6]. The electrochemical properties of Li-ion batteries depend on the electrode materials which play a major role as a key component [7,8,9,10,11]. Thus, developing a suitable and high-performance electrode material is of special significance. For many decades, carbon materials have been used in Li-ion batteries, and graphite is especially used as an electrode material, with a theoretical capacity of 372 mAhg^−1^ (LiC_6_, one lithium per six carbons) [12,13]. Despite the successful use of graphite anodes, there are some disadvantages of graphite such, as a high cost and low storage capacity (low lithium storage per carbon weight) [14], which hinder their potential use as a perfect anode. To resolve the issue of alternatives to graphite, discovering novel carbonaceous materials and their successful utilization as anodes for Li-ion batteries being great potential for the next generation of Li-ion batteries [14,15].

Recently, biomass-derived carbon anodes have been explored in various studies and used for the fabrication of Li-ion batteries. Biomass-derived activated carbon has the advantages of environmental friendliness and economic value, which make it suitable for use as an anode for Li-ion batteries. [16,17]. Furthermore, biomass-derived carbon with a unique structure can provide a suitable carbon substrate for designing high-performance batteries and it possesses an intrinsically desirable molecular structure which is essential for charge storage and transport [18,19]. Activated carbon using biomass can be synthesized by carbonizing and pyrolysis methods, and the pyrolysis of biomass at a temperature of ~1000 °C is the most common and simplest technique to obtain activated carbon [20]. Various biomass materials have been widely used for the synthesis of activated carbon; these biomass sources include wood, [21,22], orange peel, [23,24] grass, [25,26], mangosteen peel, [27] apricot shell, [28] coconut husk [29,30], rice husk [31,32,33], pomelo peel [34], pinecone shell [35], and hazelnut shell [36]. The activated carbon obtained using these biomasses has shown excellent electrochemical properties as electrode materials for energy applications due to its high conductivity, chemical and physical durability, and surface chemistry. These features of activated carbon make it a suitable choice as an anode in Li-ion batteries, offering a high power density in addition to high energy density [37,38]. Regardless of the use of biomass-derived activated carbon from the above-mentioned sources, the research for activated carbon-based anode materials still requires further investigation, as the cyclic stability and capacity performance could not be achieved. Researchers have reported a high capacity value of 1000 mAhg^−1^ for 150 cycles resulting from walnut shell-derived carbon as an anode material [39,40]. Therefore, activated carbon, which can provide a higher capacity along with long cycle stability, is desired. 

Despite the fact that rice husk has been used for the synthesis of activated carbon in various reports, the novelty of this work is in the preparation method of activated carbon using a modified chemical route with microwave irradiation. To the best of our knowledge, microwave irradiation was not used for the preprocessing of carbonization followed by activation. It is well known that microwave heating has the advantages of fast heating, energy efficiency, ease of control, a small thermal inertia and selective heating [41,42]. Generally, in the process of carbonization, the precursor is carbonized at lower temperatures (<800 °C) under an inert atmosphere, which helps in releasing the volatile gases and results in black colored char constituted mainly of carbon, followed by activation. In this work, we used microwave heating of the precursor prior to the carbonization process, which could help in enhancing the carbonization and activation by increasing the reaction rate. The activation process has a significant impact on the performance of the AC, such as the pore structure and adsorption capacity.

In this work, an activated carbon-based anode for Li-ion batteries was synthesized from rice husk, which offers little or almost no economic cost benefit as a waste byproduct. The structural, morphological and electrochemical properties of the activated carbon prepared in this study using a microwave irradiation-assisted carbonization process were characterized in detail. The electrochemical studies show that the anodes based on activated carbon from rice husk provided excellent performance in the form of a high capacity and long cyclic stability for 400 cycles, which prove to be a promising alternative to the current graphite anode and the commonly used biomass-derived anodes.

## 2. Experimental Details

### 2.1. Fabrication of Activated Carbon

All of the reagents involved in the experiments were of analytical grade and used as received without any purification. For the typical synthesis of activated carbon, rice husk (from brown rice) was obtained from a local market and cleaned by washing, then dried to perform the grinding in a grinder which resulted in the fine powder with a size of a few microns. The chemical activation process was carried out, in which the grinded powder of rice husk was placed into a domestic microwave oven at a power of 900 W for 5 min, and then heated in a muffle furnace to undergo carbonization at a temperature of 550 °C at a heating rate of 15 °C/min for 1 h. Then, the sample was cooled down under a nitrogen atmosphere at a flow rate of 120 cm^3^/min. After the carbonization process, the rice husk powder was mixed with the potassium hydroxide in a ratio of 1:4 and grinded in an agate mortar for 5 min. Then, the heating was performed again by placing the mixed powder in a muffle furnace and heating at 850 °C for 1 h at a heating rate of 15 °C/min under a nitrogen atmosphere at a flow rate of cm^3^/min. Finally, the product was washed several times with DI water to remove the impurities, followed by drying at 80 °C for 12 h.

### 2.2. Materials Characterization

To study the structural properties of the as-prepared material, an X-ray diffractometer (Phillips X’pert; MPD 3040, EA Almelo, The Netherlands) equipped with Cu Kα radiations was used with a diffraction angle range of 5–80°. The morphology of the prepared product was studied by using field-emission scanning electron microscopy (FESEM; JEOL, JSM-7600F) and transmission electron microscopy (TEM; JEOL, JEM 2100 F) operated at 200 kV. To further study the structural features of the sample, Raman spectroscopy was performed by using a confocal Raman microscope (LabRAM, HR800) with a wavelength of 633 nm and a power of 20 mW at room temperature. To measure the specific surface area, nitrogen adsorption/desorption studies were carried out using a Micromeritics ASAP 2020 system.

### 2.3. Cell Fabrication and Electrochemical Characterization

The activated carbon-based anode was prepared by mixing activated carbon with carbon black (carbon super P, MTI) and polyvinylidene fluoride (PVDF, Sigma Aldrich, MW~534,000, St. Louis, MO, USA) in a ratio of 8:1:1, respectively. After the mixture preparation, copper foil was used to cast the slurry using the doctor blade coating method followed by drying at 80 °C for 12 h. As a counter electrode, lithium foil (Sigma Aldrich, thickness = 0.75 mm, width = 45 mm, 99.9% trace metal basis) was used. A polypropylene membrane (25 μm, Celgard 2325) separator and 1 M lithium hexafluoro phosphate (LiPF_6_) dissolved in ethylene carbonate (EC) and dimethyl carbonate (DMC) (1:1 in vol %) (all from Sigma Aldrich, 99.9%) were used as an electrolyte. The coin cells (2032-type) at room temperature were assembled in an Ar atmosphere glovebox. For the battery characteristics of the fabricated cell, a multi-channel battery tester (LAND) was used and galvanostatic charge–discharge and cycling studies were carried out in a potential range between 0.0 and 2.5 V at a current density of 100 mAg^−1^. Prior to the battery cycling, an aging time of 12 h was used. In order to perform electrochemical impedance spectroscopies (EIS), a potentiostat (Biologic SP-300) was used with a perturbation of amplitude of 10 mV in the frequency range of 200 kHz to 100 mHz. Cyclic voltammetry measurements were performed at a scan rate of 20 mV s^−1^ in a potential window of 0.0 to 2.5 V.

## 3. Results and Discussion

**Figure 1:**
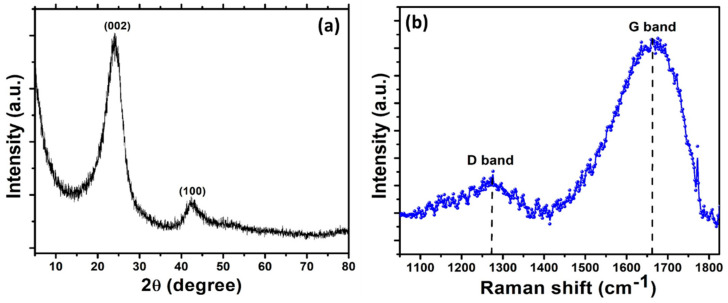
(**a**) XRD patterns and (**b**) Raman spectrum of activated carbon prepared using rice husk.

The crystalline structure of the activated carbon derived from the rice husk was studied by using XRD analysis. Figure 1a depicts the XRD patterns of the activated carbon, in which two peaks at 2θ of 24.29° and 42.62° were observed. The peak at 24.29° is ascribed to the plane (002), while the peak positioned at 42.62° corresponds to the (100) plane of graphitic structures [43]. Within the detection limit of XRD, no other phases were detected, thus indicating the successful formation of activated carbon from biomass. Normally, the intensity of the diffraction peak in the XRD pattern reveals the nature of the product to be crystalline or amorphous. A high intensity peak indicates the high crystallinity, while a broad peak shows that the product is of amorphous nature [43]. The broadness of the peak in the XRD pattern of activated carbon, as shown in Figure 1, implies that activated carbon derived from rice husk possess the defective structure of disordered carbon [44].

To further elucidate the internal structure, including the degree of crystallinity and disordering of the activated carbon derived from rice husk, Raman spectroscopy analysis was carried out at room temperature. Figure 1b shows the room temperature Raman spectrum of the activated carbon in the wavenumber range of 1000–2000 cm^−1^. Two clear bands at wavenumbers of 1274 cm^−1^ and 1660 cm^−1^ were observed, which correspond to the D and G bands, respectively [45]. The band named as D, also known as the disordered band, resulted from the dangled sp^2^ bonds, while the G band arises due to pristine carbon atoms that form activated carbon. Generally, the degree of amorphousness and/or presence/absence of defects inside the carbon materials could be observed qualitatively in the Raman spectrum. To quantify this, the ratio of the D band to the G band could provide information on the degree of amorphousness. More specifically, the higher the degree of graphitic crystallinity, the lower the intensity ratio and the lesser the defects [46]. The Raman spectrum in Figure 1b, a ratio (I_D_/I_G_) of 0.8 for activated carbon was obtained, which reveals the formation of activated carbon with some defects or having some degree of amorphous nature [47]. Normally, the carbons prepared from biomass are amorphous in nature due to their requirements of a high crystallization temperature (>1000 °C) for a few hours; however, other carbons are difficult to recrystallize together [48]. The activated carbon in this work obtained from rice husk showing a lower degree of amorphous nature, which is somewhat distinct from the commonly prepared activated carbon. In this work, a ratio of I_D_/I_G_ = 0.8 for the activated carbon was observed, which would be advantageous for charge storage applications. 

**Figure 2:**
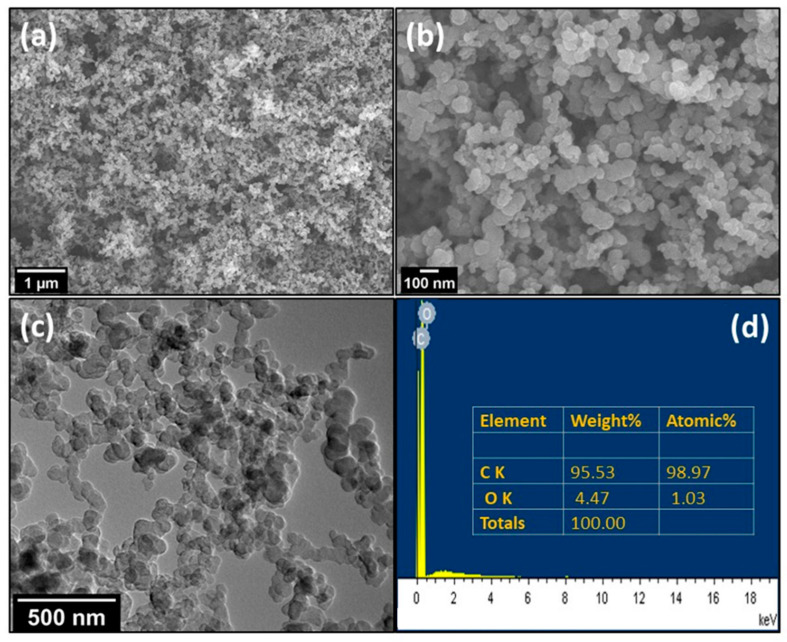
FESEM images of activated carbon at (**a**) low magnification and (**b**) high magnification. (**c**) TEM image of activated carbon and (**d**) EDX spectrum of activate carbon.

FESEM and TEM analyses were used for the morphology and structural studies of the activated carbon prepared using rice husk. The FESEM micrographs of the activated carbon at two different magnifications are shown in Figure 2a,b. It could be observed from Figure 2a that the activated carbon has a spherical particle-like morphology, with two populations of particles (smaller and bigger) of a size ranging from 40 to 90 nm. These particles have a porous nature and are densely grown all over the surface.

Further studies of morphological features of activated carbon were performed by using TEM measurements. Figure 2c shows the TEM micrograph of activated carbon, which exhibits that activated carbon are formed in the shape of nanoparticles having a size ranging from 45–95 nm. The elemental studies of the as prepared activated carbon were conducted through Energy Dispersive X-ray (EDX) analysis. Figure 2d depicts the EDX spectrum of activated carbon, it is clear from Figure 2d that the activated carbon is composed of ~98.97% carbon and 1.03% oxygen. The higher concentration of carbon component indicates that the activated carbon prepared in this method from rice husk is free from impurity. 

**Figure 3:**
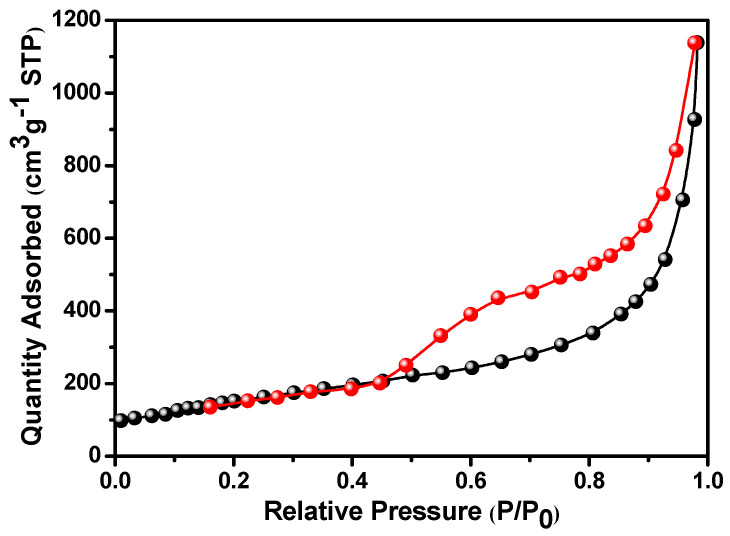
Nitrogen adsorption/desorption isotherms of rice husk-derived activated carbon.

In order to explore the specific surface are of the activated carbon prepared using rice husk, nitrogen adsorption/desorption studies were performed, and the isotherms obtained are shown in Figure 3. The Brunauer–Emmett–Teller (BET) specific surface area of rice husk-derived activated carbon was found to be ∼1962.32 m^2^ g^−1^.

**Figure 4:**
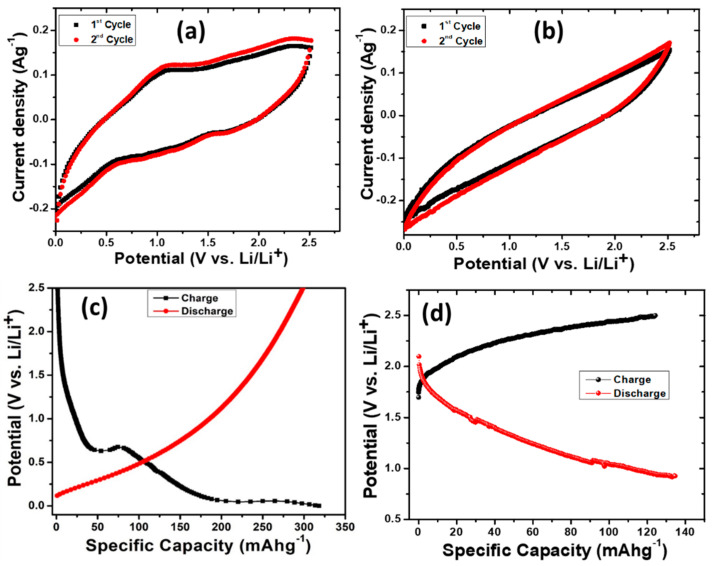
Cyclic voltammograms of (**a**) activated carbon, (**b**) commercial activated carbon at a scan rate of 20 mVs^−1^ for the first and second cycle. Galvanostatic charge–discharge curves for the first cycle of fabricated cell with (**c**) activated carbon anode, and (**d**) commercial activated carbon at a current density of 100 mAg^−1^.

Cyclic voltammetry (CV), as a valued electrochemical study, was performed for the cell containing activated carbon anode in a potential range of 0.0 to 2.5 V vs. Li/Li^+^ as shows in Figure 4a. It is clear from the CV plot that for the 1st cycle of cathodic scan, peaks positioned at ~0.52 V and 1.50 V are observed, which are suggestive of single-phase Li insertion [49,50]. Furthermore, a single peak at 1.06 V in the successive anodic scan is observed. Second cycle of CV analysis was also performed, and it was observed there are well-defined cathodic and anodic peaks which are maintained at constant redox potentials with minor changes in the current density. 

To compare the electrochemical performances of the activated carbon, CV studies of commercial activated carbon were also carried out, as shown in Figure 4b. By comparing the CV studies, significant changes are observed in the magnitude of the cathodic and anodic peak currents for the rice husk-derived activated carbon and commercial activated carbon. The activated carbon derived from rice husk possessed a higher current amplitude compared to the commercial activated carbon, revealing a higher redox activity. 

Figure 4c shows the electrochemical performance of the Li-ion batteries with activated carbon as an anode. The galvanostatic charge–discharge curves of the fabricated Li-ion batteries with the activated carbon as an anode were recorded for the first cycle in the voltage range of 0.0 to 2.5 V at a current density of 100 mAg^−1^. It is clear from Figure 4c that the capacity of the fabricated cell with an activated carbon anode is observed to be 321.52 mAhg^−1^ for the discharge cycle, and a capacity of 301.65 mAhg^−1^ is observed for the charge cycle. During the charge–discharge process, as shown in Figure 4c, a gradual curve of the activated carbon anode shows that the storage of Li-ions occurs gradually and consistently over its operating potential. Obviously, the presence of amorphousness in the activated carbon anode favors the even distribution of potentials at which Li ion insertion is electrochemically favorable, thus acting as an intrinsically safer anode [51]. Generally, there are various mechanisms of Li-ion storage of disordered carbon which include intercalation, adsorption, cavity/pore filling, surface/interface storage, and heteroatom/functional group contribution [52,53,54,55,56]. Furthermore, the gravimetric capacity of disordered carbon is significantly exceeded compared to graphite (LiC_6_), due to the contributions from different mechanisms. In this work, the improvement is achieved through the engineering of the microstructure and defect content in activated carbon; this might increase the number density of Li+ ion trapping sites, which could improve Li+ ion charging and intercalation. Furthermore, it could be inferred that carbon vacancies might be produced which can provide Li+ charging sites. Therefore, the formation of these sites might enhance Li+ ion absorption during charge and discharge cycles.

To further compare the storage ability and Li ion interactions, charge/discharge studies at a similar current density of 100 mAg^−1^ for the commercial activated carbon was conducted. Figure 4d depicts that the commercial activated carbon provided a discharge capacity of 134 mAhg^−1^ and a charge capacity of 125 mAhg^−1^. By comparing the capacities, rice husk-derived activated carbon shows a capacity of ~2.4-fold higher than that of the commercial activated carbon.

**Figure 5:**
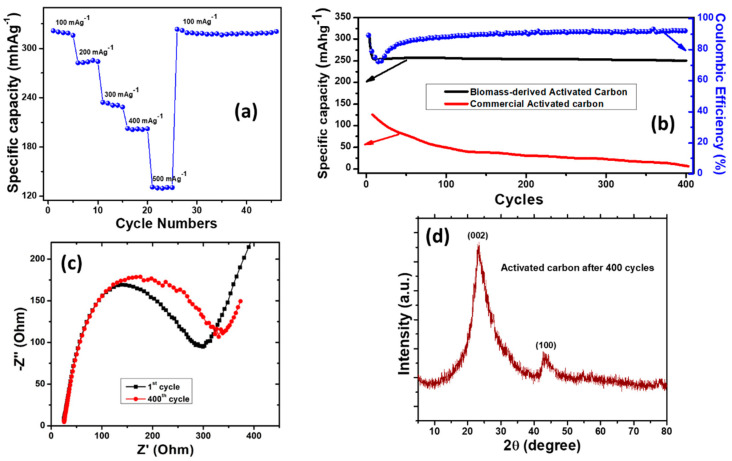
(**a**) Rate capability of activated carbon at various current densities. (**b**) Cyclic performance of the fabricated cell with the activated carbon anode and commercial activated carbon at a current density of 100 mAg^−1^. (**c**) EIS Nyquist plots of activated carbon electrode for the 1st cycle and 400th cycle. (**d**) XRD patterns of rice husk-derived activated carbon after 400 cycles of charge/discharge at a current density of 100 mAg^−1^.

The rate capability performance is one of the key factors for practical uses of an anode material for the fabricated cell. The rate performance test was carried out for cells at various current densities from 100 to 500 mAg^−1^ for five charge/discharge cycles with an identical discharge and charge current density. Figure 5a shows the rate capability performance of rice husk-derived activated carbon. It is clear that with the current density increasing from 100 to 200 mAg^−1^, a decrease in the capacity from ~321 to ~284 mAhg^−1^ was observed. A further increase in the current density to 300, 400, and 500 mAg^−1^ resulted in a decrease in capacity to 228, 202, and 130 mAhg^−1^, respectively. After five cycles at 500 mAg^−1^, when reverted to a current density of 100 mAg^−1^, the cell retained its capacity and stayed stable for the next 20 cycles. Therefore, the rate capability performance results revealed that the rice husk-derived activated carbon anode has good electrochemical properties and stability.

In order to evaluate the cyclic stability of the fabricated cell, the cycling performance of the Li-ion batteries with activated carbon as the anode was studied at 100 mAg^−1^ and is shown in Figure 5b. It can be clearly seen that during the first 10 cycles, the discharge capacity of the cell decreased from ~321 mAhg^−1^ to ~253 mAhg^−1^, and was then maintained at this capacity for 400 cycles. The gradual decrease in the capacity for the initial cycles is ascribed to Li ion consumption for solid electrolyte interface (SEI) formation. This long cyclic performance of the fabricate cell with an activated carbon anode implies that rice husk-derived carbon provides 0.68 lithium per six carbons reversibly. While the capacity of ~253 mAhg^−1^ is lower than that of the theoretical capacity of graphite (372 mAhg^−1^), the long cyclic stability of this activated carbon anode makes it a unique and potential candidate for the electrode, with reversible lithium insertion/desertion into/from the rice husk-derived carbon. Furthermore, a very stable capacity retention of ~81% and coulombic efficiency of 92.3% after 400 cycles can be maintained. 

The cyclic performance of commercial activated carbon was also investigated and compared with the rice husk-derived activated carbon, as shown in Figure 5b. Commercial activated carbon provides a capacity of ~50 mAhg^−1^ with a capacity retention of 37% at a current density of 100 mAg^−1^ for 100 cycles, which is five-fold lower than the capacity obtained for the rice husk-derived activated carbon for similar cycles. Upon further cycling, commercial activated carbon shows a capacity of ~7 mAhg^−1^ for 400 cycles with a retention of 4%, which is much lower than activated carbon using rice hus, thus, rice husk-derived activated carbon has superior stability. The discharge capacity of ~253 mAhg^−1^ for 400 cycles at a current density of 100 mAg^−1^ obtained in this work is promising, because a commonly used anode obtained from biomass usually delivers a discharge capacity of less than 300 mAhg^−1^ [57]. However, most of the studies focused on the improvement of the capacity of the Li-ion batteries based on biomass-derived anodes. In our work, the overall performance of the batteries, including the capacity and long cycle stability, has been improved with the rice husk-derived activated carbon anode. In earlier research [58], a reversible capacity of 181 mAhg^−1^ for 200 cycles at a current density of 200 mAg^−1^ was obtained from pomelo peel-derived activated carbon. In another study [44], researchers found that a banana peel-mediated activated carbon anode delivered a capacity of 200 mAh g^−1^ at 40 mAg^−1^ current density for only up to 10 cycles. Furthermore, coconut oil-based activated carbon produced a discharge capacity of 250 mAh g^−1^ at 100 mAg^−1^ for 90 cycles [59]. The capacity in this reported work is almost similar to our work and was studied at similar electrochemical conditions; however, due to the long cyclic performance obtained in our work for 400 cycles and the capacity being maintained for this long duration, our anode is unique and differs from the commonly reported biomass-based anode.

Electrochemical impedance spectroscopy (EIS) measurements are useful for studying the kinetics and interfacial behaviors of electrodes. Figure 5c shows the EIS spectrum of the Li-ion batteries with activated carbon as an anode. The intersection of the X-axis and the semicircle are used to calculate the ohmic resistance between the electrode and the electrolyte [60]. As shown in Figure 5c, the Ohmic resistance of the activated carbon-based anode was found to be 24.96 Ω for the 1st cycle; however, a slightly higher value of 25.45 Ω was observed for the 400th cycle, respectively. Furthermore, the diameters of the semicircles that are ascribed to the charge transfer resistance were found to be ~394.31 Ω for the 1st cycle and 433.30 Ω for the 400th cycle, respectively. 

In order to analyze the stability of nanomaterials, post-reaction characterizations are highly significant [61,62]. The stability of the rice husk-derived activated carbon anode was studied by performing XRD analysis. Figure 5d shows the XRD patterns of activated carbon after completing 400 cycles of charge/discharge. No obvious changes were observed in the characteristic peaks of carbon compared with the activated carbon anode before the charge/discharge cycle (Figure 1a). However, the peak width was found to be decreased slightly compared to the activated carbon pre-reaction, which indicates a slight increase in size while maintaining a similar crystal structure. Therefore, the rice husk-derived activated carbon anode shows excellent stability even after 400 cycles.

## 4. Conclusions

In summary, rice husk-derived activated carbon has been successfully synthesized and used as an anode for Li-ion batteries with enhanced performance and cyclic stability of the cell. Activated carbon using rice husk as biomass was prepared using a chemical method. Structural studies performed using XRD and Raman analyses showed the successful formation of activated carbon with some amorphous nature but with a high carbon content and free from impurities. Raman results showed the characteristics D and G bands which confirmed the formation of activated carbon. Electrochemical studies showed that the activated carbon anode delivered a discharge capacity of ~321 mAhg^−1^ at 100 mAg^−1^ and retained a capacity of ~253 mAhg^−1^ after 400 cycles. Furthermore, the capacity retention of 81% and coulombic efficiency of ~92% after 400 cycles at a current density of 100 mAg^−1^ were found to be maintained. Thus, the fabricated Li-ion batteries using activated carbon anode showed promising results in achieving a stable operation of the Li-ion batteries even at long cycles of 400, which might open a door to fabricate Li-ion batteries with biomass-derived activated carbon anodes exhibiting improved performance and cycle life.

## Data Availability

Data is available based on reasonable request.
